# Comparison of intermittent screening (using ultra-sensitive malaria rapid diagnostic test) and treatment (using a newly registered antimalarial pyronaridine-artesunate—PYRAMAX®) to standard intermittent preventive treatment with sulfadoxine-pyrimethamine for the prevention of malaria in pregnant women living in endemic areas: ULTRAPYRAPREG

**DOI:** 10.1186/s13063-022-06884-8

**Published:** 2022-11-28

**Authors:** Vivi Maketa, Japhet Kabalu, Melissa Kabena, Flory Luzolo, Hypolite Muhindo-Mavoko, Henk D. F. H. Schallig, Kassoum Kayentao, Petra F. Mens, Pascal Lutumba, Halidou Tinto

**Affiliations:** 1 Department of Tropical Medicine, University of Kinshasa (UNIKIN), Kinshasa, Democratic Republic of the Congo; 2 Amsterdam University Medical Centres, Academic Medical Centre at the University of Amsterdam (AMC), Laboratory for Experimental Parasitology, Amsterdam Institute for Infection and Immunology, Amsterdam, Netherlands; 3 Malaria Research and Training Center (MRTC), University of Sciences of Techniques and Technologies of Bamako (USTTB), Bamako, Mali; 4 Institut de Recherche en Sciences de la Santé – Clinical Research Unit of Nanoro (IRSS-CRUN), Ouagadougou, Burkina Faso

**Keywords:** Malaria, Pregnancy, IPTp-SP, ISTp, Malaria indicators, Ultra-sensitive RDTs

## Abstract

**Background:**

Intermittent preventive treatment in pregnancy (IPTp) with sulfadoxine-pyrimethamine (SP) is an important malaria control strategy in sub-Saharan Africa. Indeed, it overcomes the risk of misdiagnosis due to low peripheral parasitemia during pregnancy by treating women with SP on predetermined schedules. However, over time, the spread of *Plasmodium*-resistant strains has threatened this strategy in many countries. As an alternative, the intermittent screening and treatment for pregnancy (ISTp) aims at a monthly screening of pregnant women, preferably by using very sensitive tests such as ultrasensitive rapid diagnostic tests (us-RDTs) and the treatment of positive cases with artemisinin-based combination therapy (ACT) regardless of the presence of symptoms. Unlike IPTp-SP, ISTp prevents overuse of antimalarials limiting the drug pressure on parasites, an advantage which can be potentiated by using an ACT like pyronaridine-artesunate (Pyramax®) that is not yet used in pregnant women in the field.

**Methods:**

This study aims to compare the non-inferiority of ISTp using us-RDTs and Pyramax® versus IPTp-SP on malaria in pregnancy through a randomized clinical trial performed in Kisenso, Kinshasa, the Democratic Republic of the Congo, a malaria perennial transmission area.

**Discussion:**

The results will be essential for the National Malaria Control Program to update the malaria prevention policy in pregnant women in the Democratic Republic of the Congo.

**Trial registration:**

ClinicalTrials.gov NCT04783051

## Administrative information

Note: the numbers in curly brackets in this protocol refer to SPIRIT checklist item numbers. The order of the items has been modified to group similar items (see http://www.equator-network.org/reporting-guidelines/spirit-2013-statement-defining-standard-protocol-items-for-clinical-trials/).

**Table:** 

Title {1}	Comparison of intermittent screening (using ultra-sensitive malaria Rapid Diagnostic test) and treatment (using a newly registered antimalarial Pyronaridine - Artesunate – Pyramax®) to standard intermittent preventive treatment with sulfadoxine-pyrimethamine for the prevention of malaria in pregnant women living in endemic areas. ULTRAPYRAPREG
Trial registration {2a and 2b}.	ClinicalTrials.gov ID: NCT04783051
Protocol version {3}	Upyrapreg_ paper draft Version 1.2_ 08042022
Funding {4}	This project is a part of the EDCTP2 program supported by European Union (Grant number TMA2019CDF-2699-ULTRAPYRAPREG) and Novartis.
Author details {5a}	Vivi Maketa1, Japhet Kabalu1,2, Melissa Kabena1, Flory Luzolo1, Hypolite Muhindo-Mavoko1, Henk D. F. H. Schallig2, Kassoum Kayentao3, Petra F. Mens2, Pascal Lutumba1, Halidou Tinto4 Author’s Affiliations: 1 Department of Tropical Medicine, University of Kinshasa (UNIKIN), Kinshasa, Democratic Republic of the Congo 2 Amsterdam University Medical Centres, Academic Medical Centre at the University of Amsterdam (AMC), Laboratory for Experimental Parasitology, Amsterdam Institute for Infection and Immunology, Amsterdam, Netherlands 3 Malaria Research and Training Center (MRTC), University of Sciences of Techniques and Technologies of Bamako (USTTB), Mali 4 Institut de Recherche en Sciences de la Santé – Clinical Research Unit of Nanoro (IRSS-CRUN), Burkina Faso.
Name and contact information for the trial sponsor {5b}	University of Kinshasa Rector : Jean Marie Kayembe Ntumba jm.kayembe@unikin.ac.cd
Role of sponsor {5c}	The role of the sponsor is to design and perform the clinical trial, to analyze and interpret data, and ensure the results dissemination through reports and publication. The funders’ aim is to provide the funds necessary for all the activities in the field but they had no role in study design or field data collection. The funders will not be involved in the analyses or interpretation of the data.

## Introduction

### Background and rationale {6a}

Malaria is a significant health threat for pregnant women and their offspring in endemic settings [1, 2]. Indeed, *Plasmodium falciparum* (*Pf*) can be sequestered in the placenta during pregnancy, resulting in low peripheral parasitemia. As a consequence, standard malaria diagnostic tests can be false negative in pregnant women who actually do have a *Pf* malaria infection [3, 4].

Intermittent preventive treatment of pregnant women (IPTp) with sulfadoxine-pyrimethamine (SP) is one of the World Health Organization (WHO)’s recommended malaria prevention strategies in pregnant women in sub-Saharan African countries [2]. The IPTp-SP strategy overcomes the potential misdiagnosis of malaria in pregnant women by treating them systematically with at least three doses of SP during antenatal care (ANC) visits [5]. It can be administered from the beginning of the second trimester until delivery, provided that doses are given 1 month apart. However, in many countries, the spread of *P. falciparum* SP-resistant strains now threatens the efficacy of the IPTp-SP and can lead to the proliferation of placental-resistant parasites in pregnant women [6–10].

As an alternative for IPTp-SP, intermittent screening and treatment for pregnancy (ISTp) is considered as an option [11]. The latter entails monthly screening of pregnant women with a malaria rapid diagnostic test (RDT) and treatment of positive cases with an artemisinin-based combination therapy (ACT) regardless of the presence of symptoms. The ISTp relies on the performance of the diagnostic tests and this strategy is currently jeopardized due to false negative *P. falciparum* histidine-rich *Pf*HRP2-based RDT, the most commonly used diagnostic test to support this approach [12, 13]. The *Pf*HRP2-based RDTs have been found to detect around 45% of the PCR-positive infections in paucigravidae and about 30% in multigravidae, thereby allowing the majority of infections to persist in the placenta [14]. To circumvent this limitation, the use of ultra-sensitive RDTs (us-RDTs), which have a higher sensitivity than conventional RDTs [11, 14], is proposed as an alternative as these can avoid false negative test results [15].

Unlike IPTp-SP, ISTp limits the overuse of antimalarials and, thus, reduces drug pressure on malaria parasites [11]. This advantage could be potentiated by using an ACT that is currently not yet broadly used or should not be used by the national malaria control program for treating other strata of the population than pregnant women. Pyronaridine-artesunate (Pyramax®), a newly approved antimalarial drug is the ideal candidate for this purpose. Indeed, Pyramax®, approved for use in malaria-endemic countries since 2015, is now used in the field to treat malaria in children and adults [16].

This study hypothesized that the ISTp performed with the us-RDT and, if positive, followed by Pyramax® treatment (ISTp-US-Py) is non-inferior to IPTp-SP for the prevention of maternal malaria, maternal anemia, spontaneous abortions or intrauterine death during pregnancy, fetal morbidity (premature birth, low birth weight, small for gestational age), and neonatal mortality at childbirth.

### Objectives {7}

#### Main objective

The main objective of the study is to assess that the proportion of maternal malaria, maternal anemia, and their impact on the offspring is non-inferior when using ISTp-US-Py compared to IPTp-SP.

### Secondary objectives

The secondary objectives are to assess that:

During pregnancy:

- The proportion of asymptomatic/symptomatic malaria cases is not higher when using ISTp-US-Py compared to IPTp-SP
- The proportion of the parasite densities is not higher when using ISTp-US-Py compared to IPTp-SP
- The proportion of anemia is not higher when using ISTp-US-Py compared to IPTp-SP
- The incidence of spontaneous abortions or intrauterine deaths is not higher when using ISTp-US-Py compared to IPTp-SP

At birth:

In women:

- The proportion of asymptomatic/symptomatic malaria cases is not higher when using ISTp-US-Py compared to IPTp-SP
- The parasite densities are not higher when using ISTp-US-Py compared to IPTp-SP
- The proportion of anemia is not higher when using ISTp-US-Py compared to IPTp-SP

In the offspring including:

- Intrauterine death
- The fetal morbidities (preterm birth, low birth weight) are not higher when using ISTp-US-Py compared to IPTp-SP

During the 28-day period following the birth:

- The neonatal and early neonatal mortality of the offspring is not higher when using ISTp-US-Py compared to IPTp-SP

### Trial design {8}

This is a 2-arm randomized (1:1 ratio) non-inferiority trial. A unique registration number will be associated with a randomization list number, generated before the start of the study and which will determine the assignment of study participants to one of the study arms.

## Methods: participants, interventions, and outcomes

### Study setting {9}

The study is carried out in DRC where malaria transmission is intense and perennial.

The study is conducted in the “Maternité Esengo,” a 106-bed health facility, located in Kisenso, Kinshasa, where there is an average of 100 deliveries per month. A previous study performed in a similar setting showed that the prevalence of malaria in pregnant women was 30% [4].

Pregnant women will be recruited during ANC visits, regardless of their parity.

### Eligibility criteria {10}

Inclusion criteria (all should be present):

1. Gestation ≥16 weeks
2. Age: ≥18 years
3. Residence within the health facility catchment area
4. Willingness to adhere to study requirements and to deliver at the health facility
5. Willingness to provide written informed consent; if the woman is illiterate, she can choose an impartial witness, not related to the study, to accompany her during the consent process and they will both sign the informed consent form

Exclusion criteria:

1. Known history of allergy to SP or to an ACT
2. An ongoing antibiotic prophylaxis with cotrimoxazole
3. Current issue requiring hospital admission (including severe malaria as defined by WHO) [17]
4. Pregnancy at high risk
5. Women before 16 weeks of pregnancy will not be included because there is insufficient data on the safety of Pyramax® during the first trimester of pregnancy. However, if the WHO changes the recommendations, women in the first trimester could be treated with ACT. In this case, a modification of this protocol will be done accordingly and subjected to ethical review and to competent authorities

Participants will be excluded in case of withdrawal of the informed consent, if they take antimalarial or an antibiotic with antimalarial activity other than those prescribed by the study clinician.

### Who will take informed consent? {26a}

Pregnant women will be recruited during ANC visits, regardless of their parity and the informed consent will be obtained by the MD of the study team on the field.

### Additional consent provisions for collection and use of participant data and biological specimens {26b}

It is mentioned in the informed consent form (ICF) that, in addition to the blood collected to perform tests in the field, blood spot will be collected for further examination.

### Interventions {6b}

#### Explanation for the choice of comparators

This study will compare the IPTp-SP strategy used in routine by the NMCP with the ISTp-US-Py to assess the non-inferiority of the second strategy for the prevention of maternal malaria, maternal anemia, spontaneous abortions or intrauterine death during pregnancy, fetal morbidity (premature birth, low birth weight, small for gestational age), and neonatal mortality at childbirth.

Pyramax® was the perfect drug to use for this procedure as it has been recognized by the NMCP as a drug that can be used during pregnancy in particular although the drug is not used in the overall population.

#### Intervention description {11a}

The ISTp-US-Py group will comprise pregnant women who will be screened monthly from the beginning of the 2nd trimester with us-RDT and who will be treated with Pyramax® if the test is positive.

Pyronaridine-artesunate (Pyramax®, Shin Poong Pharmaceutical Company, South Korea) is a film-coated tablet containing 180 mg of pyronaridine tetraphosphate and 60 mg of artesunate. As part of the EDCTP2 program supported by the European Union (RIA2017MC-2025-Pyrapreg), Shin Poong (Korea) agreed to donate us treatment for this study as well. The IPTp-SP group will be pregnant women who will receive SP as recommended by the National Malaria Control Program (NMCP) at weeks 16, 28, 32, and 36 of their pregnancy. SP (Fansidar®, Roche Laboratories, Switzerland) is a drug used for the treatment of uncomplicated malaria in children and adults and for IPTp. Fansidar is presented as scored tablets containing 500 mg of sulfadoxine and 25 mg of pyrimethamine. The drug continues to be beneficial to both mother and baby, even in areas of SP resistance [18].

#### Concomitant therapies

Any medication or other therapy taken by the study participant will be recorded.

#### Criteria for discontinuing or modifying allocated interventions {11b}

Changing the distribution of intervention is not possible in the framework of this study. In the event of an allergy or other adverse event (AE) that may impact participants’ health or that of the offspring, the participant will be removed from the study and will no longer receive the intended drug in the arm in which they were allocated.

#### Strategies to improve adherence to interventions {11c}

In order to ensure compliance with the protocol by the study personnel, two monitoring visits are planned throughout the study. The latter will focus on the review of source documents, the distribution of participants by study arm, and the accountability of the study drugs and tests.

#### Relevant concomitant care permitted or prohibited during the trial {11d}

It will be advised to the participants to return to the health center if they feel unwell to receive a concomitant therapy.

#### Provisions for post-trial care {30}

Coverage of care after the end of the study’s planned follow-up period is scheduled up to 28 days after delivery, though this is not a part of the study but rather for ethical reasons.

#### Outcomes {12}

##### Primary outcome

The primary outcome will be the assessment of the malaria status over the pregnancy at every visit for each study participant.

##### Secondary outcomes

During pregnancy, the following outcomes will be assessed at every visit for each study participant: the anemia status, the incidence of spontaneous abortions, and the intrauterine deaths.

At birth, the outcomes will be assessed as follows in the offspring: the fetal morbidity and the intrauterine death.

Within the 28 days of post-partum, the following outcomes will be assessed in the infant: the early neonatal mortality and the neonatal mortality.

### Participant timeline {13}

Pregnant women fulfilling the inclusion/exclusion criteria will be recruited during the ANC visit over an enrollment period of about 20 months. At every scheduled and unscheduled visit, the study team will collect vital signs, blood pressure, weight, data on the medical history since the last visit (including any treatment taken), information on any AE, and current signs and symptoms (if any). At the same time, the team will collect blood samples for malaria diagnosis (us-RDT, thick and thin blood film), Hb, and dried blood spots.

The outcome of pregnancy, birth weight, APGAR score, and maternal Hb will be collected as soon as possible after delivery. A placental blood sample will be collected for the diagnosis of malaria (thin blood film) and dried blood spots. The newborn will be examined for congenital abnormalities and will be reassessed again at day 28 (Table 1).

**Table 1:** Time and event schedule

	Enrolment	Allocation								
Timepoint**	-t_**1**_	0	M_**1**_	M_**2**_	M_**3**_	M_**4**_	M_**5**_	M_**6**_	Delivery	4–6 weeks post-end of pregnancy
**Enrolment**										
**Eligibility screen**	X									
**Informed consent**	X									
**Allocation**		X								
**Interventions**										
***IPTp-SP***			X	X	X	X	X	X		
***ISTp***			X	X	X	X	X	X		
**Assessments**										
***Mother***										
***Temperature***		X	X	X	X	X	X	X		
***Thick smear***		X	X	X	X	X	X	X		
***TDR, US-TDR***		X	X	X	X	X	X	X		
***Hb***		X	X	X	X	X	X	X		
***Mother***										
***Temperature***			X	X	X	X	X	X	X	
***Thick smear***			X	X	X	X	X	X	X	
***TDR, US-TDR***			X	X	X	X	X	X	X	
***Hb***			X	X	X	X	X	X	X	
***Offspring***										
***Weight, temperature***									X	
***APGAR***									X	
***Thick smear***									X	
***Hb***									X	
***Newborn assessment***										X

### Sample size {14}

The calculation of the sample size has performed using the Sealed Envelope Ltd. 2012. Power calculator for binary outcome non-inferiority trial. [Online] Available from: https://www.sealedenvelope.com/power/binary-noninferior/.

Referring to the results of a study comparing IPTp-SP and IST (dihydroartemisinin-piperaquine) [11], the number of participants required to detect a difference of 10% with a significance level of 5% and a power of 80% to assess the non-inferiority of IST to IPTp-SP vary as described in Table 2. Variables of interest considered were the malaria status, the anemia status, and the low birth weight rate.

**Table 2:** Variation of the sample size according to the difference of the outcome of interest

	IPTp	IST	Sample size
Low birth weight	10.5	15.7	122
Malaria infection	18.9	23.4	196
Anemia	30	25	220

*NB*: IST with dihydroartemisinin-piperaquine and standard RDT was used as an example to calculate the sample size for the current study as there are no previous studies could be found that include assessment of Pyramax® and us-RDT for use as IST.

### Recruitment {15}

Based on the assumption described above, a total of two hundred and twenty pregnant women is the minimal sample size required to answer our research question. However, anticipating on a non-compliance and/or loss-to-follow-up rate of 10%, the adjusted total sample size required will be 242 pregnant women (121 participants per arm).

### Assignment of interventions: allocation

#### Sequence generation {16a}

The study participants will be assigned to the groups using a simple, not stratified randomization of a 1:1 ratio. The randomization list will be generated by an independent data technician using Excel Microsoft prior to the inclusion.

#### Concealment mechanism {16b}

The randomization numbers will be stored in sealed individual envelopes that will be opened in front of the participants.

#### Implementation {16c}

The randomization list will be generated by a data manager non-involved in the study.

### Assignment of interventions: blinding

#### Who will be blinded {17a}

All the participants will be aware of the drug regimen they will be assigned to. But the statistician will be blinded for the analysis to avoid any bias.

#### Procedure for unblinding if needed {17b}

Not applicable. The study team and the participants themselves will all be aware of the arms where participants will be included.

### Data collection and management

#### Plans for assessment and collection of outcomes {18a}

Pregnant women fulfilling the inclusion/exclusion criteria will be recruited during ANC in the study site over a period of about 20 months. Data will be entered by the MDs in paper CRF designed by the study site in accordance to variables to collect over the participants’ visits during the study. Later, data managers will be in charge to enter the data collected in papers in the electronic database. To ensure the accuracy of the process, source data verification of every entry will be performed by the study supervisors before the validation of the database.

At every scheduled and unscheduled visit, the study team will collect vital signs, blood pressure, weight, data on the medical history since the last visit (including any treatment taken), information on any AE, and current signs and symptoms (if any). At the same time, the team will collect blood samples for malaria diagnosis (us-RDT, thick and thin blood film), Hb, and dried blood spots for PCR.

The outcome of pregnancy, birth weight, APGAR score, and maternal Hb will be collected as soon as possible after delivery. Dried blood spots will be collected on placental blood. The newborn will be examined for congenital abnormalities. Both the mother and the newborn will be reassessed at day 28.

##### Demographic data and medical history

Demographic data and a general history of past and/or present illnesses, the medical history since the last visit (including any treatment taken), information on any AE, and current signs and symptoms (if any) will be recorded.

##### Physical and clinical examination

Vital signs (body temperature, pulse rate), blood pressure, and weight will be measured.

An obstetrical examination will be performed and the fetal viability will be recorded.

- PCR

Bloodspots of 50 μl will be collected on filter paper (Whatman grade 3) at each visit and subsequently every time a blood slide is done. Samples will be used to determine sub-microscopic parasitemia.

- Malaria diagnostic

In addition to an us-RDT detecting circulating *P. falciparum* antigens, thick and thin blood films will be prepared, dried, and stained with Giemsa according to standard operating procedures (SOP).

If positive, the parasite density (PD) will be calculated by counting the number of *P. falciparum* parasites per 200 leukocytes based on the actual number of WBC/μl. In case this information is missing, the PD will be estimated assuming WBC of 8000/μl. The PD per microliter will be calculated using the following formula:

$$\mathrm{Parasites}/\mathrm{\mu}\mathrm{l}(\mathrm P/\mathrm{\mu}\mathrm{l})=\frac{\mathrm{Number}\;\mathrm{of}\;\mathrm p\mathrm a\mathrm r\mathrm a\mathrm s\mathrm i\mathrm t\mathrm e\mathrm s\;\mathrm c\mathrm o\mathrm u\mathrm n\mathrm t\mathrm e\mathrm d\times8,000}{\mathrm N\mathrm u\mathrm m\mathrm b\mathrm e\mathrm r\;\mathrm o\mathrm f\;\mathrm l\mathrm e\mathrm u\mathrm k\mathrm o\mathrm c\mathrm y\mathrm t\mathrm e\mathrm s\;\mathrm c\mathrm o\mathrm u\mathrm n\mathrm t\mathrm e\mathrm d}$$

The thin smear will be examined for species determination.

Blood smears will be independently read by 2 qualified microscopists and the mean of the PD will be used in the study. Further details are described and explained in the SOP (SOP: quality control (QC) guidelines for the laboratories participating in the study).

### Plans to promote participant retention and complete follow-up {18b}

Participant’s visits are scheduled on monthly basis. In case of a missing visit, the site clinicians will be in charge to phone the participants to enquire for the reason of the missing visit and encourage them to come to the study site. In case participants cannot be reached by phone, a community health worker will be visiting the households for enquiring and encourage participants to come to the study site. In case participants are not reachable, they will be considered as lost to follow-up after 3 missing visits and all attempts to reach out to them will be documented.

In case of loss to follow-up, all the data collected during the woman participation in the study will be used for the analysis. However, in case of consent withdrawal, the participant data will not be used for the analysis.

### Data management {19}

#### Data management and storage

Data management will be handled by the University of Kinshasa which has the required expertise in data management of clinical trials and epidemiological studies.

Data entry and review will be performed following the Data Entry Guidelines and the Data Management plan. Besides this central management, two monitoring visits are planned to check the information entered into the electronic CRF against the source documents available on site. Any modification done onto the electronic CRF will be automatically registered. The final database will be obtained after the resolution of all queries and will be locked for statistical analysis to be carried out according to a pre-established data analysis plan that will be developed by a statistician and submitted for comments and advice to the Trial Steering Committee (TSC).

Every patient will have a personal source document file. Data will be collected onto the source document and entered later in an electronic CRF. Data will be entered on a daily basis on the database.

Study documents will be archived for 10 years and granted to the sponsor for trial-related monitoring, audits, DSMB, and Ethical Committee review and regulatory inspections when applicable.

### Confidentiality {27}

Collected personal information will be restricted to meet the objectives of the study. Prior to the study start, all study staff will sign a confidentiality agreement form. Every participant will be assigned a unique study identification code and no name or personal information will appear in the database or will ever be published. All personal information mentioned in the signed informed consent forms and other documents will be kept under lock and will only be available to the project coordinator and data managers. Information stored in the database will be protected by unique usernames and passwords, which will only be available to minimum appropriate authorized personnel.

### Plans for collection, laboratory evaluation, and storage of biological specimens for genetic or molecular analysis in this trial/future use {33}

For this study, the laboratory analysis carried out will be the Hb dosage using a Hemocue, a diversified evaluation of malaria by TDR, US-TDR, thick and thin smear, and PCR.

Hb assay, TDR, US-TDR, and blood smear will be done on-site. Blood will be taken from a dried blood spot for further PCR analysis. The dried blood spot will be stored in individual zippers, each coded for the study, stored in a dry place and containing silica gel to prevent spoilage.

## Statistical methods

### Statistical methods for primary and secondary outcomes {20a}

#### General analysis principles

Data analysis will be performed after the end of the follow-up period of the last participant included in the study.

Study participants will receive two different interventions depending on which group they are enrolled in. The first group will undergo intermittent screening during pregnancy using ultra-sensitive malaria rapid diagnostic test and treatment with pyronaridine-artesunate—Pyramax® (ISTp-US-Py), and the second group will receive an intermittent preventive treatment as suggested by the NMCP. The analysis will mainly compare the two groups to assess the outcomes of the study. All AEs (severe or non-severe) will be reported in the trial publication and compared in the study arms and gestational weeks. To assess the effect of the study interventions in the IPT and the ISTp arms, we will use the intention-to-treat approach. Meaning that all the participant who has been randomized to an arm or another will be considered as part of that arm for the analysis despite their level of completion.

Data from every woman included with baseline and at least one further visit will be analyzed in the treatment arms to which they were assigned. Safety data from all participants who received at least one dose of the study drug will be used in the analysis to describe AEs. AEs will be grouped by treatment group for the number of events and number of participants with type of event. Descriptive statistics will be summarized by treatment arm and visits.

For all statistics, the confidence intervals will be two-sided and at the 95% level, and the value of *p* < 0.05 will be interpreted as statistically significant.

### Participant flow

The number of participants screened, included, randomized, withdrawn, and lost to follow-up will be summarized in the analyses.

### Baseline comparability of the study groups

Baseline data will be summarized by arm. Categorical variable data will be summarized by number and percentage per category. The synthesis of continuous variables will be performed using the mean and standard deviation for approximately normally distributed variables. For variables that are not normally distributed, the median and interquartile ranges will be used. At this point, no formal statistical testing will be performed because differences between study arms are more likely to be the result of chance than the consequence of randomization.

#### Study outcomes

##### Primary outcome

The primary outcome will be the assessment of asymptomatic/symptomatic malaria status and the parasite density of *P. falciparum* during pregnancy at every visit for every participant. The asymptomatic malaria status is defined as the presence of *P. falciparum* diagnosed by an us-RDT in the peripheral blood and a T°<37.5°C; symptomatic malaria is defined as the presence of *P. falciparum* diagnosed by an us-RDT in the peripheral blood and temperature and/or a history of temperature T°≥37.5°C. The parasite density is assessed by the quantification of *P. falciparum* parasites in the peripheral blood of participants by a thin blood smear examined by standard malaria microscopy.

##### Secondary outcomes

During the pregnancy, the anemia status and the incidence of spontaneous abortions or intrauterine deaths will be evaluated in each visit for every study participant. Anemia is assessed as a level of hemoglobin (Hb) <11g/dl. The spontaneous abortion is the loss of pregnancy naturally before 20 weeks of gestation. Intrauterine death refers to the loss of pregnancy after 20 weeks of gestation.

At birth, for the offspring, the neonatal, the early neonatal, and the neonatal morbidity will be assessed. The neonatal morbidity is estimated at birth and refers to any of the following: reterm birth (birth before 37 weeks of gestation), low birth weight (birth weight under 2500 g), and small for age. Early neonatal and neonatal morbidities will be assessed respectively as infant death at birth or within 7 days of life and infant death within the first 28 days of life.

### Interim analyses

Not applicable. The non-inferiority of ISTp-US-Py compared to IPTp-SP will be assessed after the follow-up period of the last participant.

### Methods for additional analyses (e.g., subgroup analyses) {20b}

Not applicable. The aim of this study will be to assess the non-inferiority of ISTp-US-Py compared to IPTp-SP. We will carry out the evaluation by comparing the performance of ISTp-US-Py and IPTp-SP. No sub-group analysis will be performed.

### Methods in analysis to handle protocol non-adherence and any statistical methods to handle missing data {20c}

For each variable, statistical tests will be performed with the available data. A 10% sample size adjustment will be made to ensure adequate power despite the loss to follow-up of some participants.

### Plans to give access to the full protocol, participant-level data, and statistical code {31c}

The essential element of the protocol has been registered in an accessible clinical trial register clinicaltrials.gov: NCT04783051. The final results of this trial will be published in open-access journals. All the anonymized participant data will be also published as additional tables along with the publications.

### Oversight and monitoring

#### Composition of the data monitoring committee, its role, and reporting structure {21a}

The trial steering committee will be composed of independent members of the Faculty of Medicine of the University of Kinshasa who are not related to the study. The steering committee will be responsible for ensuring the progress of the trial, the compliance with the protocol, and the safety of study participants. Members will have virtual or in-person meetings to review AE reports and monitor reports at quarterly meetings.

The ULTRAPYRAPREG study is embedded in a larger PYRAPREG study (PACTR202011812241529); the Data Safety Monitoring Board (DSMB) of the PYRAPREG study will be in charge of the ULTRAPYRAPREG data monitoring.

#### Adverse event reporting and harms {22}

All AEs, both severe and non-severe, will be collected and reported in the participants’ CRF and in a database. In the event of serious AEs, the study team will report to the national ethics committee and the trial steering committee no later than 48 h after the team becomes aware of the event.

An AE will be defined as any adverse medical event in a subject, regardless of the possibility of a causal relationship. The study team will collect the AEs after the consent is signed and the participant enrolled in the study. If a participant experiences an AE after enrollment but before receiving the study intervention, the event will be reported as unrelated to the study products of investigation.

All AEs occurring after entry and before the end of the follow-up period will be recorded until the end of the AE.

A SAE is any untoward medical event that results in any of the following: life-threatening condition (i.e., immediate risk of death), severe or permanent disability, prolonged hospitalization, or significant danger as determined by the Data Safety Monitoring Board. SAEs occurring after a subject has been withdrawn from the study will not be reported unless the investigators believe the event may have been caused by a study product of investigation. Investigators will determine the relationship between an event and the study drug based on a temporal relationship to the study drug, as well as whether the event is unexpected or unexplained given the subject’s clinical course, previous medical conditions, and concomitant medications.

All AEs, both severe and non-severe, will be collected and reported in the participants’ CRF and in a database. In the event of serious AEs, the study team will report to the national ethics committee and the trial steering committee no later than 48 h after the team becomes aware of the event.

#### Frequency and plans for auditing trial conduct {23}

There are two monitoring planned for the trial. The two visits will aim to ensure the accuracy of the CRF completion and the informed consent process. The monitoring will be conducted by an independent monitor from the University of Kinshasa not related to the study.

#### Plans for communicating important protocol amendments to relevant parties (e.g., trial participants, ethical committees) {25}

Before any protocol amendment is performed on the field, it will first have to be approved by the National Ethics committee and the sponsor. At the end of the study, a final report will be shared to the National Ethics committee.

#### Dissemination plans {31a}

The project will be accessible via the Pyrapreg and UNIKIN sites to improve its visibility.

The results of the project will be shared with the National Malaria Control Program to update the policy for the prevention of malaria in pregnant women. The results shared during will be shared during conferences, congresses, and workshops. Results will be published in peer-reviewed open-access journals.

## Discussion

This protocol will assess the proportion of maternal malaria, maternal anemia, spontaneous abortions or intrauterine death during pregnancy, fetal morbidity, and neonatal mortality at childbirth in ISTp-US-Py compared to IPTp-SP. WHO recommends a package of interventions for preventing malaria and its adverse effects during pregnancy, which include the IPTp-SP as a prevention strategy to control malaria in sub-Saharan countries [5]. SP depends on the doses and ANC coverage, but in addition to that, the strategy is now threatened by the spread of *Plasmodium*-resistant strains. The resistance to SP undermines the ability of IPTp-SP to minimize the adverse effects of malaria in pregnancy [19], necessitating alternative approaches [20]. The potential approach is the ISTp which depends on the generation of the rapid diagnostic test and the ACT used. Moreover, the pooled analysis of five trials provides further evidence that ISTp with the current generation of RDTs is not superior to the existing strategy of IPTp with SP [21].

The us-RDT used in this trial has a higher sensitivity [22] than the conventional ones and the ACT used is newly approved as the antimalarial in DRC. The effective and safe treatment is needed to protect a pregnant woman with malaria, which is the objective of this project. At our knowledge, this study is the first to use the combined us-RDT in the ISTp strategy and Pyramax® as treatment. Pregnant women are a vulnerable population and require special attention. For that, the outcome of this study will be relevant in settings where malaria in pregnancy has a high morbidity and where the SP resistance is emerging. If successful, the findings can be the evidence-base for the update of malaria control policy by the NMCPs.

## Trial status

Date of beginning: 6 May 2021

Recruitment has already been completed, but the submission is done before the last patient/last visit. The submission was not done earlier because we were too immersed in the fieldwork and the exchanges with the co-authors took time because of the busy schedule of each of them.

## Abbreviations

AMC: Academic Medical Centre
ANC: Antenatal care
ACT: Artemisinin-based combination therapy
CRF: Case report form
DSMB: Data Safety and Monitoring Board
DRC: Democratic Republic of the Congo
EDCTP: European & Developing Countries Clinical Trials Partnership
GMP: Good manufacturing practice
Hb: Hemoglobin
ICF: Informed consent form
IRSS: Institut de Recherche en Sciences de la Santé
IPTp-SP: Intermittent preventive treatment for pregnant women with sulfadoxine-pyrimethamine
ISTp: Intermittent screening and treatment for pregnancy
ISTp-US-Py: Intermittent screening in pregnancy using ultra-sensitive malaria rapid diagnostic test and treatment with pyronaridine-artesunate—Pyramax®
NMCP: National Malaria Control Program
*Pf*: *Plasmodium falciparum*
PD: Parasite density
PCR: Polymerase chain reaction
QC: Quality control
RDT: Rapid diagnostic test
SP: Sulfadoxine-pyrimethamine
SOP: Standard operating procedures
T°: Temperature
us-RDTs: Ultrasensitive RDTs
UNIKIN: University of Kinshasa
WHO: World Health Organization
WBC: White blood cell
μl: Microliter
